# Supplementary material for: The interactome of intact mitochondria by cross-linking mass spectrometry provides evidence for coexisting respiratory supercomplexes
Source: Mol Cell Proteomics. 2017 Dec 8;17(2):216–32. doi: 10.1074/mcp.RA117.000470 (PMC5795388; doi:10.1074/mcp.RA117.000470)
Supplement: Supplemental Data [file supp_RA117.000470_133922_0_supp_23978_fzffwf.zip › spectra_annotation/mito_spectra_annotation/200-1-4-1-2-1.pdf]

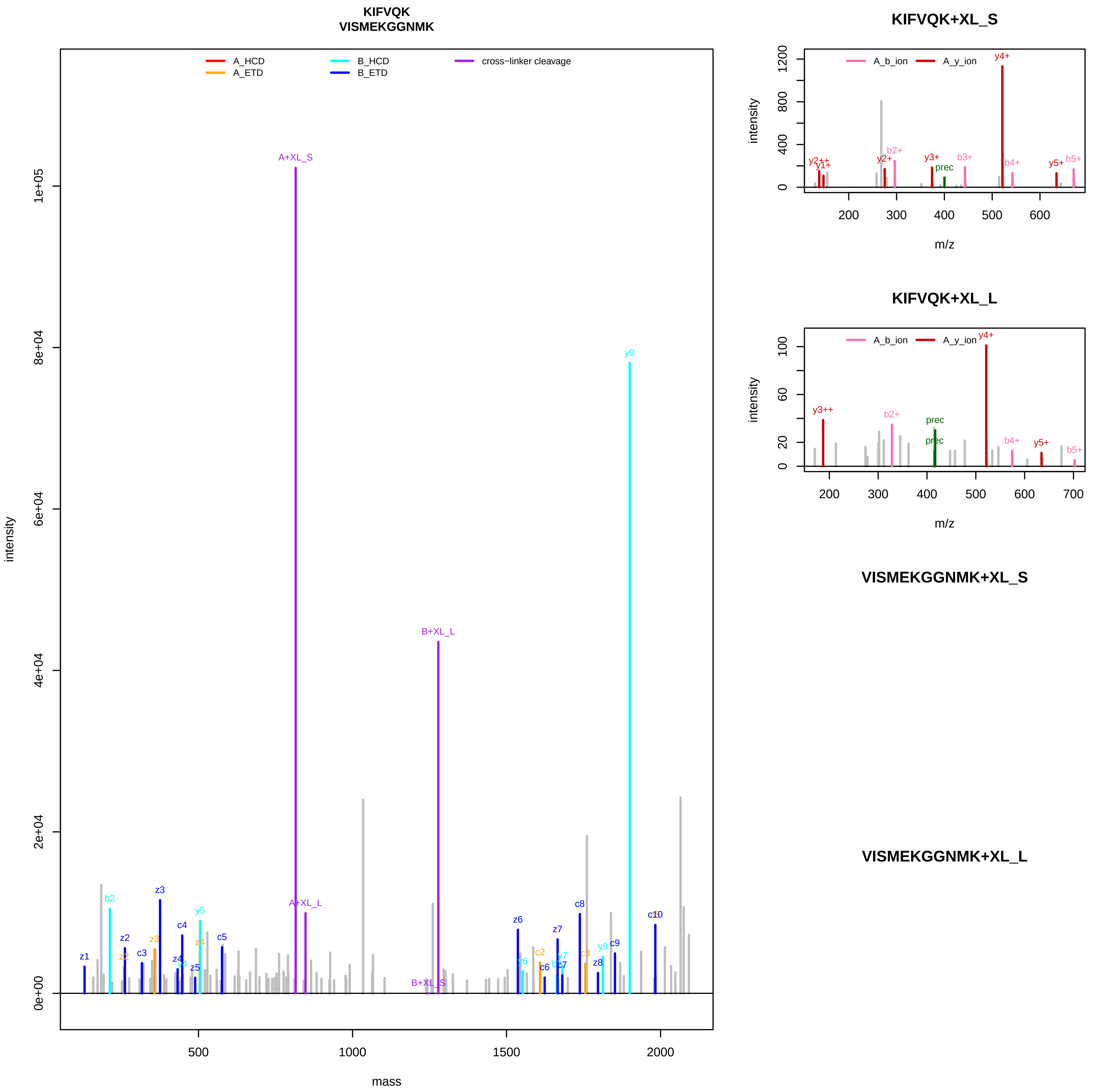
KIFVQK
VISMEKGGNMK
A_HCD
A_ETD
B_HCD
B_ETD
cross−linker cleavage
intensity
mass
A+XL_S
A+XL_L
B+XL_L
B+XL_S
y9
b2
z1
z2
c3
z3
c4
z4
y4
z5
y5
c5
z6
y6
c2
c6
z7
y7
c7
c8
c3
z8
y8
c9
c10
KIFVQK+XL_S
A_b_ion
A_y_ion
y2++
y1+
b2+
y2+
y3+
prec
b3+
y4+
b4+
y5+
b5+
m/z
KIFVQK+XL_L
y3++
VISMEKGGNMK+XL_S
VISMEKGGNMK+XL_L
